# Deep Medullary Vein Asymmetry and Clinical Outcomes in Patients with Ischemic Stroke and Successful Endovascular Treatment

**DOI:** 10.3390/jcm15103813

**Published:** 2026-05-15

**Authors:** Giorgio Busto, Francesco Arba, Simone Ferretti, Mattia Tripari, Guido Fanfani, Giovanni Noto, Andrea Lastrucci, Angelo Barra, Alessandro Fiorenza, Sara Mancini, Cosimo Nardi, Davide Gadda, Andrea Ginestroni, Enrico Fainardi

**Affiliations:** 1 Neuroradiology Unit, Department of Radiology, Careggi University Hospital, 50134 Florence, Italy; guido.fanfani@unifi.it (G.F.); giovanni.noto@unifi.it (G.N.); alessandro.fiore993@gmail.com (A.F.); saraman85@gmail.com (S.M.); gaddad@aou-careggi.toscana.it (D.G.); a.ginestroni@gmail.com (A.G.); enrico.fainardi@unifi.it (E.F.); 2 Stroke Unit, Careggi University Hospital, 50134 Florence, Italy; francesco.arba@unifi.it (F.A.); simone.ferretti@unifi.it (S.F.); mattia.tripari@unifi.it (M.T.); 3 Department of Allied Health Professions, Careggi University Hospital, 50134 Florence, Italy; lastruccia@aou-careggi.toscana.it (A.L.); barraa@aou-careggi.toscana.it (A.B.); 4 Radiodiagnostic Unit n. 2, Department of Experimental and Clinical Biomedical Sciences, University of Florence, 50121 Florence, Italy; cosimo.nardi@unifi.it

**Keywords:** ischemic stroke, deep medullary vein, collaterals, multiphase CT-angiography, endovascular treatment

## Abstract

**Background:** Deep medullary vein (DMV) drainage has been suggested as a new biomarker for predicting clinical outcomes in patients with acute ischemic stroke (AIS). We evaluated this hypothesis in patients who received endovascular treatment (EVT) within 24 h of symptom onset. **Methods:** We performed a retrospective study of consecutive AIS patients at a single institution treated with EVT achieving successful recanalization (final mTICI score ≥2b). DMV drainage was graded on a three-point scale (0-1-2) during the second peak venous phase of mCTA by assessing contrast filling, with grade 2 indicating a favorable DMV profile. Our primary outcomes were functional independence, defined as a modified Rankin Scale (mRS) score of 0–2 at 90 days, and ordinal mRS shift at 90 days. Secondary outcomes were excellent clinical status (mRS 0–1 at 90 days), hemorrhagic transformation, and symptomatic intracranial hemorrhage. We investigated independent associations using multivariable logistic and ordinal regression analyses as appropriate, adjusting for age, sex, baseline mRS, NIHSS at onset, occlusion site, intravenous thrombolysis, onset-to-CT time, and ASPECTS. **Results:** We included 506 patients; the mean age was 76 years. A favorable DMV profile was present in 394 (78%) patients. We found that DMV doubled the odds of achieving functional independence (OR = 2.22; 95% CI = 1.28–3.85) and was associated with a shift towards better functional outcomes in ordinal regression analysis (cOR = 1.93; 95% CI = 1.24–3.02), whereas we did not find any association between a favorable DMV profile and secondary outcomes. **Conclusions:** In AIS patients successfully recanalized with EVT, a favorable DMV profile was associated with better functional outcomes. Further investigations may clarify the clinical use and predictive ability of this novel radiological marker.

## 1. Introduction

Acute ischemic stroke (AIS) occurs when blood flow is abruptly interrupted in cerebral arteries [1]. Reperfusion therapy, such as intravenous thrombolysis and/or endovascular treatment (EVT), showed efficacy in reducing the burden of stroke in terms of disability and social costs [2]. Several imaging biomarkers at baseline can be used to prognosticate the tissue fate, helping clinicians in decision-making and improving outcome prediction [3]. Along with early ischemic changes detectable on plain computed tomography, pial collaterals have been suggested as a pivotal factor in predicting functional outcomes and final infarct volume [4]. However, the collateral network does not only rely on arterial collaterals, suggesting that other vascular pathways can play a key role in prognosis [5]. Venous outflow has recently gained increasing interest due to its ability to improve the adequacy of arterial leptomeningeal inflow and blood transit through ischemic tissue, thereby maximizing the effectiveness of reperfusion therapies [6]. In particular, deep medullary veins (DMVs) have been shown to promote efficient venous outflow from deep brain tissue by facilitating the removal of metabolic waste and maintaining optimal cerebral blood flow [7,8]. In addition, DMVs can be easily assessed because of their recognizable anatomical architecture, which is perpendicular to the lateral ventricles. Susceptibility-weighted magnetic resonance imaging (MRI) sequences have been used to establish DMV drainage by exploiting the susceptibility effect of deoxyhemoglobin in these veins [7]. However, multiphase CT-angiography (mCTA), a time-resolved technique, may aid in identifying DMV drainage with an easier, faster approach than MRI, relying on the second-peak venous phase [9], and is potentially applicable in acute stroke assessment. In this study, we aimed to assess whether a favorable DMV profile, evaluated on mCTA, could optimally predict functional outcomes in patients with AIS undergoing EVT within 24 h of symptom onset.

## 2. Methods

### 2.1. Data Availability, Standard Protocol Approval, and Patient Consent

The data from this study will be available to qualified researchers upon reasonable request to the corresponding author, subject to approval by the local ethics committee. Written informed consent was obtained from each patient or their legally authorized representatives at the time of admission. This cohort study was approved by the Ethical Committee of the University of Firenze (PN 26299).

### 2.2. Patient Selection and Study Design

Data were obtained from a retrospective observational study involving a prospectively collected cohort of AIS patients with anterior circulation large-vessel occlusion treated with EVT at Careggi University Hospital in Florence, from January 2017 to December 2023. The registry’s design details have been published elsewhere [10]. For this study, all patients who underwent a multimodal CT protocol at admission—comprising non-contrast computed tomography (NCCT) and mCTA of cervical and intracranial vessels—were included. Inclusion criteria were: (i) arrival at the emergency department within 24 h of witnessed symptom onset or the last well-seen time; (ii) evidence of middle cerebral artery M1 or M2 segment occlusion or internal cerebral artery occlusion on mCTA; (iii) eligibility for EVT. Exclusion criteria included: (i) severe pre-stroke disability, indicated by a modified Rankin Scale (mRS) score of 3 or higher; (ii) low-quality NCCT or mCTA images caused by motion artifacts; (iii) inability to perform the full multi-modal CT protocol or the standard digital subtraction angiography at baseline or the 24 h follow-up NCCT.

### 2.3. Imaging Acquisition

All imaging protocols were performed using a 128-slice scanner (Philips Brilliance iCT, Best, The Netherlands). NCCT helical scans were acquired from the skull base to the vertex using the following imaging parameters: 120 kV, 337 mAs, 0.6 mm collimation, 1 s rotation, and a table speed of 15 mm/rotation, with a scan time of 5 s. The CTA of the cervical and intracranial vessels was carried out as follows: 0.7 mL/kg of contrast was used (up to 90 mL maximum), with a 5- to 10 s delay between injection and scanning. The scan settings included 120 kV, 251 mAs, and a 0.75 s rotation. Slice thickness was 0.8/0.4 mm with imbricated slices, and the total scan time was 4 s. The CTA covered from the carotid bifurcation to the vertex. The second and third phases were taken with an 8-s delay to allow repositioning of the table to the skull base. Each additional phase lasted 3.4 s. Axial images were reconstructed with overlapping 0.4 mm sections. Maximum-intensity-projection multiplanar reconstructions were generated in axial, coronal, and sagittal planes, each with a 10 mm thickness and 3 mm interval, focusing on the circle of Willis.

### 2.4. Imaging Analysis

**Figure 1:**
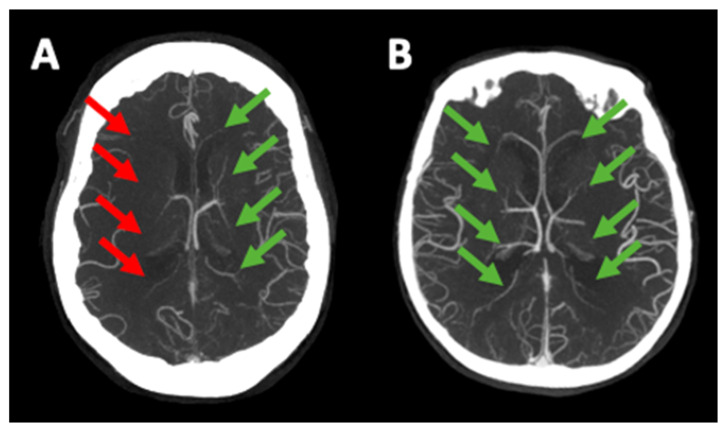
Multiphase CT-angiography comparison between unfavorable (**A**) and favorable (**B**) deep medullary vein profiles. Red arrows indicate a lack of venous opacification, while green arrows show full vein opacification.

The evaluation of early ischemic changes was performed on the baseline NCCT using the Alberta Stroke Program Early CT Score (ASPECTS) [11]. Occlusion sites were identified through mCTA and classified as terminal internal carotid artery, middle cerebral artery M1, or M2 occlusions. The arterial collateral status on mCTA was graded by two certified diagnostic neuroradiologists, G.B. and E.F., both with over 10 years of experience, who were blinded to clinical information. They used a six-point scale based on a previously published scoring system [12], where higher scores indicate better collateral circulation. DMV profiles were evaluated on the peak venous phase of mCTA by assessing contrast filling as absent (0), partial (1), or full (2) by comparing the affected side with the contralateral healthy side (Figure 1) [9]. Only grade 2 was regarded as a favorable DMV profile. Inter-reader agreement between these two neuroradiologists in evaluating acute stroke imaging features using multimodal imaging was previously assessed and found to be excellent, with a Cohen’s kappa coefficient exceeding 0.8 [13]. Any discrepancies between the two readers were discussed and resolved through consensus. Reperfusion status was determined based on the final run of digital subtraction angiography, using the modified Thrombolysis in Cerebral Infarction (mTICI) scale, where a score of ≥2b indicated successful reperfusion [14]. Hemorrhagic transformation (HT) was classified on NCCT at 24 h from symptom onset or last known well according to the Heidelberg bleeding classification [15]. Symptomatic intracranial hemorrhage (sICH) was defined as any intracranial hemorrhage associated with a ≥4-point increase in NIHSS [16]. Follow-up infarct volumes were manually segmented on 24 h NCCT scans using ITK-SNAP software (version 3.8.0-beta) by readers who were unaware of EVT results and clinical outcomes [17].

### 2.5. Outcomes of Interest

Functional outcomes were assessed using the modified Rankin Scale (mRS) at 90 days after the stroke onset. The primary outcomes were functional independence, defined as an mRS score of 0–2 and a shift on the ordinal mRS at 90 days. Secondary outcomes included: excellent outcome (mRS 0–1 at 90 days), presence of any HT, and occurrence of sICH.

### 2.6. Statistical Analysis

Continuous variables were summarized as medians (interquartile ranges [IQRs]) or means (standard deviations [SDs]), depending on their distribution, which was assessed using the Shapiro–Wilk test. The Mann–Whitney test was used for variables with non-normal distributions, while Student’s *t* test was applied for those with normal distributions. Categorical variables were presented as counts (percentages) and analyzed with the chi-square test. Baseline clinical and radiological features were described using descriptive statistics and compared between patients who attained functional independence (mRS 0–2 at 90 days post-EVT) and those who did not. Associations between DMV profiles and functional outcomes were examined using binary logistic and ordinal regression analyses. Both unadjusted and adjusted models were performed. All adjusted analyses accounted for prespecified covariates: age, sex, pre-stroke mRS, baseline NIHSS, occlusion site, intravenous thrombolysis, onset-to-CT time, collateral status, and ASPECTS. Effect sizes from these regressions are reported as unadjusted and adjusted odds ratios (ORs) and common odds ratios (cORs), with 95% confidence intervals (95% CIs). ORs indicate the odds of outcomes associated with a 1-point increase in DMV opacification (improvement of venous drainage) compared to no change, with no increase in DMV opacification serving as the reference.

All analyses were conducted using the statistical software packages SPSS version 29.0 (www.spss.com) and MedCalc 23.5.5 (www.medcalc.org). Statistical significance was set at *p* < 0.05 for a two-sided test.

## 3. Results

### 3.1. Baseline Characteristics of the Study Population

**Figure 2:**
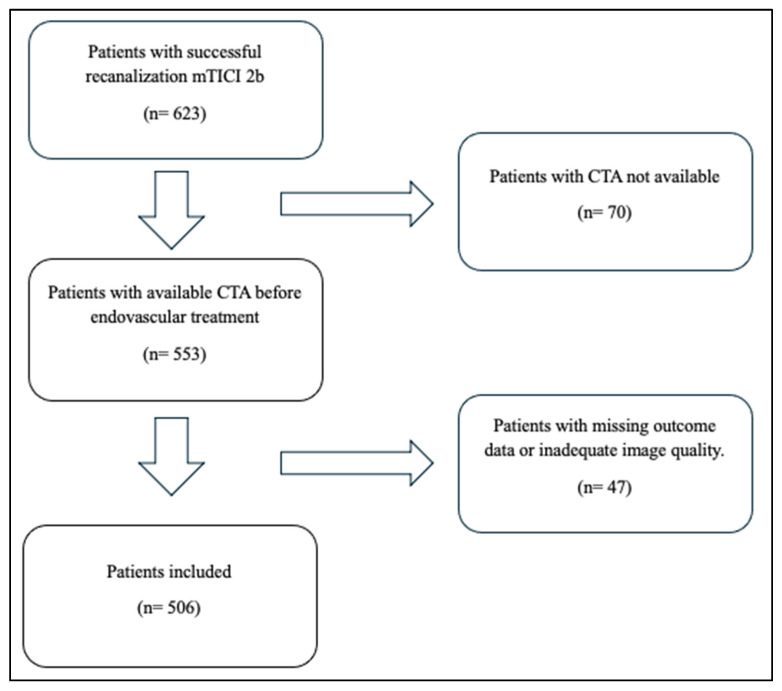
Flowchart of patient selection.

During the study period, 623 patients underwent EVT and achieved successful recanalization. Of these, 47 (8%) patients were excluded because of missing outcome data or inadequate image quality. A further 70 (11%) patients were excluded because CTA was not available prior to endovascular treatment (e.g., direct transfer to the angiography suite from another hospital). This left a total of 506 patients for the final analysis (Figure 2).

Mean (SD) age was 76.1 (12.2) years, 269 (53%) patients were male, the median (IQR) baseline NIHSS score was 18 (12–23), and the median ASPECTS was 8 (7–9). Intravenous thrombolysis was administered in 220 (44%) patients, and the median onset-to-groin puncture time was 290 min (210–450). Baseline characteristics were comparable between patients with and without favorable DMV profiles (Table 1).

### 3.2. Association Between DMV Profiles and Outcomes

**Figure 3:**
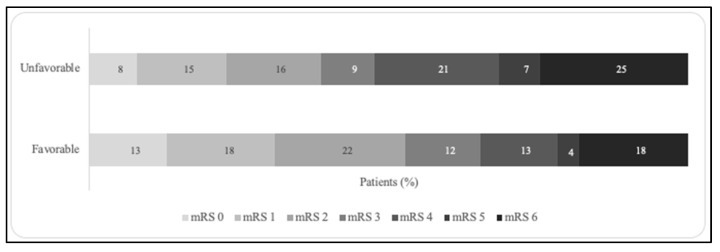
The 90-day mRS distribution stratified by deep medullary vein profiles. mRS indicates the modified Rankin scale.

**Figure 4:**
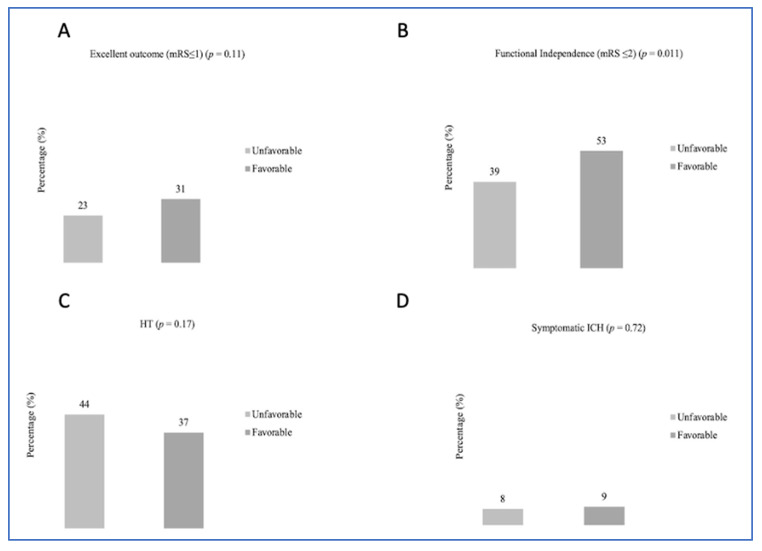
Univariate associations between deep medullary vein profiles and excellent outcome (**A**), functional independence (**B**), death (**C**), symptomatic intracerebral hemorrhage (**D**).

At 90 days, 143 (28%) patients achieved an mRS score of 0–1, 244 (48%) had an mRS score of 0–2, and 96 (19%) had died. Distribution of modified Rankin Scale (mRS) scores is shown in Figure 3. Any HT occurred in 193 (38%) patients, including 45 (9%) cases of sICH. Univariable analysis (Figure 4) showed that functional independence at 90 days was more frequent among patients with a favorable DMV profile than among those without (53% vs. 39%, *p* = 0.011). This finding was further confirmed in multivariable logistic regression (OR = 2.13; 95% CI = 1.23–3.70). There were no differences in HT (37% vs. 44%, *p* = 0.17) or sICH (9% vs. 8%, *p* = 0.72) occurrence between the two groups. In ordinal regression analysis, a favorable DMV profile was associated with a shift towards better functional outcomes (common OR = 1.82; 95% CI = 1.18–2.78) compared with subjects with an unfavorable DMV profile.

## 4. Discussion

Among AIS patients treated with EVT who successfully recanalized, we found that a favorable DMV profile (i.e., full opacification of deep medullary veins, grade 2) was associated with a higher likelihood of achieving better functional outcomes at 90 days post-EVT. However, we did not observe any association with any HT or sICH.

A poor DMV profile in AIS patients has been previously evaluated with MRI [18] and was found to be associated with HT after intravenous thrombolysis [19], poor clinical outcomes after ischemic stroke [20], and worse clinical outcomes after small vessel occlusion [21]. However, MRI is not widely available for acute stroke assessment worldwide, whereas CT is generally the preferred and most commonly used imaging technique. In a previous study, a poor DMV profile detected on mCTA was associated with poor clinical outcomes in acute ischemic stroke [22]; however, the small sample size and the need for confirmation in external cohorts limited the reliability of those findings. Our observation was based on a larger sample size and focused on patients treated with EVT who achieved successful recanalization, a subgroup more likely to have good functional outcomes. Nonetheless, we found that a favorable DMV profile doubled the likelihood of achieving functional independence, confirming and extending previous findings that show a strong association between DMV drainage and good clinical outcomes [22,23]. Ordinal shift analysis further confirmed these findings, giving consistency to this association. Although we did not find statistically significant associations with HT, it should be noted that the occurrence of any HT was higher in patients with an unfavorable DMV profile; however, sICH was also similar between the two groups, suggesting no relation between the DMV profile and clinically relevant HT. The DMV plays a pivotal role in draining blood from the deep periventricular structures of the brain, thereby contributing to metabolic clearance [24]. During brain ischemia, the presence of metabolic waste products and the mismatch between oxygen supply and demand cause the meningeal or medullary veins on the infarcted side to appear dilated compared with the healthy side of the same layer, reflecting effective collateral circulation and, consequently, a higher likelihood of a positive outcome [25]. Our results are consistent with previous findings [22,23] and expand on the value of DMV filling in patients treated with endovascular procedures who achieve successful recanalization, indicating that recanalization of the occluded vessel is only the first step towards favorable outcomes and that venous radiological markers may aid prognostic stratification.

Several venous outflow scores that assess both superficial and deep pathways have been proposed [26,27]. Interestingly, DMV drainage has not been evaluated previously and could be a promising biomarker for exploring the role of venous drainage in AIS patients. Moreover, it remains to be determined whether a comprehensive evaluation of arterial collaterals and DMV drainage could improve prognostication in subjects undergoing EVT. Notably, DMV and arterial collaterals were independently associated from each other with functional outcome in our study. This seems to suggest that DMV drainage and arterial collaterals are not epiphenomena of each other, but rather represent two different and complementary sides of the collateral network. Finally, the overall evaluation of arterial, tissue, and venous collaterals (i.e., the cerebral collateral cascade) [28,29], as well as the Multimodal Collateral Score, which considers arterial, tissue, and both superficial and deep venous collaterals [30], has shown a strong association with infarct growth, functional outcome, and final infarct lesion. DMV drainage may help clarify the role of venous outflow, and future studies are warranted to determine whether it provides additional prognostic information when evaluated together with arterial and tissue collaterals.

Our study has limitations. First, the retrospective design may introduce unmeasured bias and does not allow causal associations. Selection bias could not be excluded for patients with unavailable mCTA or missing outcome data; however, the attrition rate was less than 20% overall, lending acceptability to our study population and, therefore, to our results. Again, we could not account for other variables potentially associated with DMV drainage, such as the presence and extent of cerebral small-vessel disease, which has previously been associated with an unfavorable DMV profile [21]. In this regard, future studies could explore this association and further evaluate whether DMVs add prognostic value compared with other radiological predictors of stroke outcome, such as collateral status [31] and cerebral small-vessel disease [32,33]. We also acknowledge that CTA is not the gold standard for assessing DMV drainage and grading [7]; however, we adopted an arbitrary dichotomic classification to simplify the assessment of favorable DMV drainage and yield meaningful, biologically plausible associations. Among the strengths of the study should be noted the assessment of DMVs by two readers blinded to clinical data, the single-center design, which allowed homogeneous, standardized procedures in a real-world setting, and the appropriate sample size for the specific topic.

## 5. Conclusions

Our study showed that assessing DMV on mCTA could provide clinically relevant prognostic information to identify AIS patients who are likely to benefit from successful recanalization after EVT. Our findings deserve further confirmation in larger cohorts before implementation in clinical practice.

## Figures and Tables

**Table 1:** Baseline characteristics of the study population.

Variable	Total (n = 506)	DMVs (n = 394)	Not DMVs (n = 112)	*p*-Value
**Age, years, mean (SD)**	76.1 ± 12.2	76.0 ± 12.5	76.4 ± 11.1	0.74
**Male, n (%)**	269 (53)	211 (54)	58 (52)	0.70
**NIHSS, median (IQR)**	18 (12–23)	17 (12–22)	18 (12–23)	0.62
**mRS pre-stroke, median (IQR)**	0 (0–1)	0 (0–1)	0 (0–1)	0.78
**ASPECTS, median (IQR)**	8 (7–9)	8 (7–9)	8 (7–9)	0.17
**Blood glucose, mg/dL, mean (SD)**	129.7 ± 41.7	129.0 ± 42.3	132.1 ± 39.7	0.50
**Hypertension, n (%)**	378 (75)	296 (75)	82 (73)	0.65
**Diabetes, n (%)**	100 (20)	78 (20)	22 (20)	0.96
**Smoke, n (%)**	81 (16)	63 (16)	18 (16)	0.97
**Atrial fibrillation, n (%)**	157 (31)	120 (31)	37 (33)	0.60
**Anticoagulants, n (%)**	106 (21)	80 (20)	26 (23)	0.50
**Aspirin, n (%)**	119 (24)	97 (25)	22 (20)	0.27
**rtPA, n (%)**	220 (44)	165 (42)	55 (50)	0.13
**Time to groin, min, median (IQR)**	290 (210–450)	270 (210–390)	300 (210–464)	0.17
**Arterial collateral status, median (IQR)**	4 (3–4)	4 (3–4)	4 (3–4)	0.10
**Good collateral status, n (%)**	309 (61)	249 (63)	60 (54)	0.07
**Site of occlusion, n (%)**				0.19
**ICA**	33 (7)	30 (8)	3 (3)	
**M1-MCA**	85 (17)	68 (17)	17 (15)	
**M2-MCA**	77 (15)	56 (14)	21 (19)	
**Other (tandem occlusion/unknown)**	311 (62)	240 (61)	71 (63)	

All data are in numbers (%) unless otherwise stated. DMV = deep medullary vein; NIHSS = National Institute of Health Stroke Scale; SD = standard deviation; IQR = interquartile range; mRS = modified Rankin Scale; ASPECTS = Alberta Stroke Programme Early CT Score; rtPA = recombinant tissue plasminogen activator; ICA = internal carotid artery; M1-MCA = M1 segment of middle cerebral artery; M2-MCA = M2 segment of middle cerebral artery.

## Data Availability

The data presented in this study are available on request from the corresponding author, due to ethical restrictions.

## Abbreviations

AIS, acute ischemic stroke; DMV, deep medullary vein; mCTA, multiphase CT-angiography; EVT, endovascular treatment; MRI, magnetic resonance imaging; ASPECTS, Alberta Stroke Programme Early CT Score; mRS, modified Rankin scale.

## References

[1] Hilkens N.A., Casolla B., Leung T.W. (2024). Stroke. Lancet.

[2] Nguyen T.N., Abdalkader M., Fischer U., Qiu Z., Nagel S., Chen H.-S., Miao Z., Khatri P. (2024). Endovascular management of acute stroke. Lancet.

[3] Abdalkader M., Siegler J.E., Lee J.S., Yaghi S., Qiu Z., Huo X., Miao Z., Campbell B.C.V., Nguyen T.N. (2023). Neuroimaging of Acute Ischemic Stroke: Multimodal Imaging Approach for Acute Endovascular Therapy. J. Stroke..

[4] Maguida G., Shuaib A. (2023). Collateral Circulation in Ischemic Stroke: An Updated Review. J. Stroke.

[5] Ravindran V.A., Killingsworth M.C., Bhaskar S. (2021). Cerebral collaterals in acute ischemia: Implications for acute ischemic stroke patients receiving reperfusion therapy. Eur. J. Neurosci..

[6] Faizy T.D., Kabiri R., Christensen S., Mlynash M., Kuraitis G., Mader M.M.-D., Albers G.W., Lansberg M.G., Fiehler J., Wintermark M. (2021). Association of Venous Outflow Profiles and Successful Vessel Reperfusion After Thrombectomy. Neurology.

[7] Taoka T., Fukusumi A., Miyasaka T., Kawai H., Nakane T., Kichikawa K., Naganawa S. (2017). Structure of the Medullary Veins of the Cerebral Hemisphere and Related Disorders. Radiographics.

[8] Zhang R., Huang P., Jiaerken Y., Wang S., Hong H., Luo X., Xu X., Yu X., Li K., Zeng Q. (2021). Venous disruption affects white matter integrity through increased interstitial fluid in cerebral small vessel disease. J. Cereb. Blood Flow Metab..

[9] Chu Y., Yin Z.-X., Ni W.-J., Lu S.-S., Shi H.-B., Liu S., Wu F.-Y., Xu X.-Q. (2024). Prognostic Value of Venous Outflow Profiles on Multiphase CT Angiography for the Patients with Acute Ischemic Stroke After Endovascular Thrombectomy. Transl. Stroke Res..

[10] Busto G., Morotti A., Casetta I., Barra A., Fiorenza A., Di Pasquale F., Maccaglia M.G., Toffali M., Mancini S., Carlesi E. (2024). Hypoperfusion intensity ratio correlates with collaterals and predicts outcome and infarct volume in acute ischemic stroke patients. Eur. J. Clin. Invest..

[11] Pexman J.H.W., Barber P.A., Hill M.D., Sevick R.J., Demchuk A.M., Hudon M.E., Hu W.Y., Buchan A.M. (2001). Use of the Alberta Stroke Program Early CT Score (ASPECTS) for assessing CT scans in patients with acute stroke. AJNR Am. J. Neuroradiol..

[12] Busto G., Zini A., Gentile M., Migliaccio L., Rustici A., Simonetti L., Cardillo M.L., Casetta I., Ginestroni A., Poggesi A. (2026). Defining the Prognostic Performance of Multiphase CT-Angiography in Ischemic Stroke Patients. Eur. J. Radiol..

[13] Stone A., Jiang S.T., Stahl M.C., Yang C.J., Smith R.V., Mehta V. (2023). Development and Interrater Agreement of a Novel Classification System Combining Medical and Surgical Adverse Event Reporting. JAMA Otolaryngol. Head Neck Surg..

[14] Zaidat O.O., Yoo A.J., Khatri P., Tomsick T.A., von Kummer R., Saver J.L., Marks M.P., Prabhakaran S., Kallmes D.F., Fitzsimmons B.-F.M. (2013). Cerebral Angiographic Revascularization Grading (CARG) Collaborators; STIR Revascularization working group; STIR Thrombolysis in Cerebral Infarction (TICI) Task Force. Recommendations on angiographic revascularization grading standards for acute ischemic stroke: A consensus statement. Stroke.

[15] Zhou Y., Zhang L., Cavalcante F., Suzuki K., Treurniet K.M., Yan B., Mitchell P., Bush S., Fischer U., Kaesmacher J. (2025). Intracranial Hemorrhage in Patients With Stroke After Endovascular Treatment with or Without IV Alteplase: An Individual Participant Data Meta-Analysis. JAMA Neurol..

[16] Hacke W., Kaste M., Fieschi C., von Kummer R., Davalos A., Meier D., Larrue V., Bluhmki E., Davis S., Donnan G. (1998). Randomised double-blind placebo-controlled trial of thrombolytic therapy with intravenous alteplase in acute ischaemic stroke (ECASS II). Second European-Australasian Acute Stroke Study Investigators. Lancet.

[17] Yushkevich P.A., Pashchinskiy A., Oguz I., Mohan S., Schmitt J.E., Stein J.M., Zukić D., Vicory J., McCormick M., Yushkevich N. (2019). User-Guided Segmentation of Multi-modality Medical Imaging Datasets with ITK-SNAP. Neuroinformatics.

[18] Morita N., Harada M., Uno M., Matsubara S., Matsuda T., Nagahiro S., Nishitani H. (2008). Ischemic findings of T2-weighted 3-tesla mri in acute stroke patients. Cerebrovasc. Dis..

[19] Terasawa Y., Yamamoto N., Morigaki R., Fujita K., Izumi Y., Satomi J., Harada M., Nagahiro S., Kaji R. (2014). Brush sign on 3-T T2*-weighted MRI as a potential predictor of hemorrhagic transformation after tissue plasminogen activator therapy. Stroke.

[20] Yu X., Yuan L., Jackson A., Sun J., Huang P., Xu X., Mao Y., Lou M., Jiang Q., Zhang M. (2016). Prominence of medullary veins on susceptibility-weighted images provides prognostic information in patients with subacute stroke. Am. J. Neuroradiol. AJNR.

[21] Wang X., Lyu J., Duan Q., Li C., Huang J., Meng Z., Wu X., Chen W., Wang G., Niu Q. (2024). MR-STARS Investigators. Deep medullary vein damage correlates with small vessel disease in small vessel occlusion acute ischemic stroke. Eur. Radiol..

[22] Drozdov A.A., Javan R., Leon Guerrero C.R., Sparks A.D., Taheri M.R. (2020). Asymmetry of medullary veins on multiphase CT-angiography in patients with acute ischemic stroke. J. Stroke Cerebrovasc. Dis..

[23] Li H., Lan Y., Ju R., Zang P. (2023). Deep medullary veins as an important imaging indicator of poor prognosis in acute ischemic stroke: A retrospective cohort survey. Quant Imaging Med. Surg..

[24] Vilan A., Ribeiro J.M., Reis C., Sampaio L. (2018). Deep medullary veins and brain injury. J. Pediatr..

[25] Wijngaard I., Wermer M., Boiten J., Algra A., Holswilder G., Meijer F.J.A., Dippel D.W.J., Velthuis B.K., Majoie C.B.L.M., van Walderveen M.A.A. (2016). Cortical venous filling on dynamic computed tomographic angiography: A novel predictor of clinical outcome in patients with acute middle cerebral artery stroke. Stroke.

[26] Parthasarathy R., Kate M., Rempel J.L., Liebeskind D.S., Jeerakathil T., Butcher K.S., Shuaib A. (2013). Prognostic evaluation based on cortical vein score difference in stroke. Stroke.

[27] Adusumilli G., Faizy T.D., Christensen S., Mlynash M., Loh Y., Albers G.W., Lansberg M.G., Fiehler J., Heit J.J. (2023). Comprehensive venous outflow predicts functional outcomes in patients with acute ischemic stroke treated by thrombectomy. Am. J. Neuroradiol..

[28] Faizy T.D., Mlynash M., Kabiri R., Christensen S., Kuraitis G.M., Mader M.M., Flottmann F., Broocks G., Lansberg M.G., Albers G.W. (2022). The Cerebral collateral cascade. Neurology.

[29] Huang L., Zhang H., Li W., Gong C., Jiang S., Li Z., Yuan J., Xu T., Chen Y., Zhang L. (2025). Cerebral collateral cascade associated with infarct growth rate in ischemic stroke patients undergoing endovascular treatment. Eur. J. Radiol..

[30] Busto G., Pensato U., Casetta I., Marcello G.U., Barra A., Poggesi A., Carlesi E., Mancini S., Ginestroni A., Miele V. (2026). Comprehensive assessment of arterial, tissue, and venous collaterals for evaluating the infarct growth rate: The Multimodal Collateral Score. Ann. Clin. Transl. Neurol..

[31] Gensicke H., Al-Ajlan F., Fladt J., Campbell B.C.V., Majoie C.B.L.M., Bracard S., Hill M.D., Muir K.W., Demchuk A., San Román L. (2022). Comparison of Three Scores of Collateral Status for Their Association With Clinical Outcome: The HERMES Collaboration. Stroke.

[32] Arba F., Testa G.D., Limbucci N., Nappini S., Renieri L., Pracucci G., Nencini P., Inzitari D. (2019). Small vessel disease and clinical outcomes after endovascular treatment in acute ischemic stroke. Neurol. Sci..

[33] Arba F., Inzitari D., Ali M., Warach S.J., Luby M., Lees K.R., STIR/VISTA Imaging Collaboration (2017). Small vessel disease and clinical outcomes after IV rt-PA treatment. Acta Neurol. Scand..

